# Loot

**Published:** 1869-09-15

**Authors:** 


### LOOT.

### *Permanent Contraction of a Limb Cured by Subcutaneous Injection of Atropia.*

M. Desprez obtained a remarkable success in the case of a delicate young lady who had been for some years subject to articular rheumatism, and for a long time past had suffered from a fixed and extreme contraction of the arm, fol lowing in rheumatic inflammation of the shoulder joint. When M. Desprez first saw her, there was no remaining swelling of the joint, but the limb was somewhat atrophied from want of movement. Frictions with belladonna accomplished some, but not very much, good. A solution of sulphate of atropia was then prepared, 1 part in 400; and of this 25 drops (1-16 grain) were injected over the pectoralis major. Slight temporary intoxicative symptoms were produced. Three days later there was a marked improvement in the mobility of the joint; the muscles were less rigid, and there was less pain on attempting to move the arm. A second injection of 30 drops (1-13 grain) was made at the same point, and this was followed, at intervals, by three others of thirty-five drops each (between 1-11 and 1·12 grain), which *completed the cure.* The movement of the joint was quite restored, the atrophy has since disappeared, and the use of tonics and good feeding has subsequently much improved the patient's general health.

### *Hypodermic Injections of Morphia in the Reduction of Hernia.*

Dr. Ravoth reports (Berlin, Klin. Wochensch, Juin 7) some remarkable results from the subcutaneous injection of morphia in cases of hernia which had previously resisted the taxis. Quarter grain doses were used.

In these cases the morphia was injected in the region of the hernia, and when the patient was fully under its influence a loose towel was laid over the face and water dashed rpon it. Even without taxis it is said the hernia was sometimes reduced during the shock.

Prof. Hebra's clinic for skin diseases is mostly attended by foreigners, who pay a double fee, $15.00. He treats about 1,000 patients yearly. Male house patients are divested of their entire clothing, and pass about the class. Female patient are not required to expose the genitals.

### *Constipation in Old Age.*

For occasional use in the treatment of constipation in old age, Dr. J. M. DaCosta (*Medical and Surgical Reporter*) recommends the following formula:

| | | |
|---|---|---|
| ℞ | Podophyllin | gr. 1-20; |
| | Extract belladonna, | gr. 1-20; |
| | Capsicai, | gr. 1-4; |
| | Pulveris rhei, | gr. j. |

For one pill. To be taken three times daily. Belladonna is a stimulant to the muscular fibres of the intestines. Podophyllin is useful in torpor of the upper portion of the bowels, to increase the secretion of the liver.—*Phys. and Pharm.*

### *Treatment of Lead Poisoning.*

Dr. William Frank Smith states (*Lancet*) that in lead poisoning the treatment by iodide of potassium has been less efficacious in his practice than that by sulphuric acid and the sulphates. The formula used by him, which acts, he says, "with remarkable celerity and certainty," is as follows: sulphate of quinine, sulphate of iron, of each one grain; strychnia, 1-36 of a grain; dilute sulphuric acid five minims; sulphate of magnesia, one drachm; water, one ounce; three times a day.—*Med. Gazette.*

### *The Oxalate of Cerium in the Vomiting of Pregnancy.*

℞ Cerii oxal.; ext. lupul, *aa.* gr. xxiv. M. et. div. in pil. xii., cap. i. ter in die. At same time exhibit bromide of potassium in ten grain doses, with the tincture of yellow cinchona bark and spirits of ammonia. The mixture and pills appear to constitute a most successful plan of treatment.—*Dublin Medical Express and Circular.*

### *Sponge Tents.*

Knowing the fact that absolute or *strong* alcohol will quickly *set* the fibres of common sponge, after having been moulded or compressed into any given size or shape, I was led to the following quick and easy method of preparing sponge tents, tampons, etc.

The sponge is first thoroughly moistened with water and pressed as dry as the strength of the hand will permit; then having formed it into the desired shaps and size by the hand, or by pressing into a quill or any other tube or mould, it is immersed into the alcohol. If the spirit is sufficiently strong (90 to 100 per ct.) the sponge is *immediately* set into the given shape, which it retains perfectly after the pressure or mould is removed. It is then hard, firm and inflexible, and may be trimmed to a sharp point, or any other desired shape.

To restore it to its former size and shape it is only necessary to moisten it with a few drops of water. The alcohol sets the sponge perfectly, whether the amount of compression be much or little, so that the degree of dilitation, attainable by the use of tents thus prepared, will of course depend upon the size after moulding and the degree of pressure used. As this process of preparation works perfectly and *without* delay, its advantages are obvious.—*Boston Medical and Surgical Journal.—Cincinnati Lancet and Observer.*

***Medicated Vapors.***

Experiments are now in progress at the Neckar Hospital (Paris), and also at the Asylum for Convalescents, at Vincennes (France), upon the application of the vapor of water which has been caused to pass over various non-volatile salts, carrying with it a portion of them, to the treatment of disease. Concerning the Convalescent Asylum at Vincennes, the *Gazette des Hopitaux* reports, upon the authority of M. Dionis, that of forty thousand convalescents admitted there during the past ten years but one hundred and fifty-one have died, in the proportion of one to five hundred and twenty-six.

***Metrorrhagia.***

Dr. H. E. Gantillon reports to the *Gazette des Hopitaux* a case of metrorrhagia resulting from miscarriage at four months, which, after having resisted various modes of treatment, amongst others three applications of the curette of Recamier, and tamponing the vagina with perchloride of iron, yielding finally and completely to two intra-uterine injections of the perchloride made on the 18th of December and upon the 15th of February following.

The first injection was 15 drops of the liquid of twenty-two degrees of strength; the second twenty drops of thirty degrees. The Dr. uses a syringe of his own invention, with recurrent jets to facilitate the escape of the liquid from the uterus. While asserting that the proceeding is entirely free from danger or risk of accident, the writer acknowledges the occurrence of consecutive fever and uterine and abdominal pain, which, on each occasion of the operation, compelled the patient to remain in bed five days.

### *Solvents for Croupal Membrane.*

The *Gazette Medicia di Lombardia* records the results of many experiments on the solubility of false membrane. The specimens selected were, as nearly as possible, of the same consistence, and of a gramme in weight.

1. Solution of iodide of potassium, one in ten. At the end of fourteen hours the false membrane was reduced to filaments.
2. Sulphate of zinc, same strength. After fourteen hours membrane had shrunk.
3. Bromide of potassium. After fourteen hours membrane was transformed into a pulpy substance.
4. Chloride of sodium, chloride of barium, and hyposulphate of soda give same result.
5. Cyanide of potassium. Complete solution in fourteen hours.
6. Borax. Membrane became yellow and tough.
7. Muriate of ammonia. No result.
8. Sulphate of iron. No result.
9. Carbonate of potash. Complete solution.
10. Sulphate of soda. No result.
11. Chlorated potash. In three hours became like charpie.
12. Lime water. Same effect in same time.
13. Bicarbonate of soda. Perfect solution in three hours.
14. Nitrate of silver, one in ten. Hardening and contraction.—*Med. Press and Circular.*

### *Lead Poisoning a Cause of Vaginitis.*

Attention has of late been much drawn to the subject of vaginitis, and particularly on account of the surgical treatment inflicted upon the women suffering from this affection, and which is recommended as the only cure in such cases. The attention of the medical profession, almost dormant upon this subject since the time of Michon, was all at once attracted thereto by the recital of the cruel mutilations to which Dr. Marion Simms and several of his followers submitted the victims of this singular disease. The treatment of this fortunately very rare affection, as practised by the above very bold American gynæcologist, has found but few advocates among us, and its further practice will not be encouraged by the ætiology of the disease which comes to us from the native country of Dr. Sims.

Dr. Neftel, of New York, relates three cases of vaginitis occurring in women of high social position, in one of whom, on account of the aggravated form of the disease, sexual intercourse had become impossible, coincident with a saturnine intoxication resulting from the prolonged use of a cosmetic containing lead. The intoxication manifested itself by paralysis and atrophy of the extensors, so that the patients were unable to extend their hands, fingers or thumbs, while the supinator muscles, as well as the deltoid, biceps and triceps, were in a normal state. The electro-mnscular contractility and sensibility were very much diminished, if not entirely abolished. Two of the patients were cured by the internal use of the Iodide of Potassium and Sulphur, and without having employed any local means for the vaginitis. It disappeared at the same time with the paralysis, so that the married woman who had hitherto been sterile, and had for that cause consulted Dr. Marion Sims, has since become a mother.—*L' Union Médicale and Am. Jour. of Obstetrics.*

### *Fractures of the Clavicle.*

MM. Denonvilliers and Richet demonstrated to the clinical class, at the Children's Hospital (Paris), that fractures of the clavicle oppose no direct obstacle to the upward movement of the arm as has been believed hitherto. The movement is frequently prevented by pain, but its possibility was practically shown, by them, even in cases where the fracture was comminuted.—*Gazette des Hop.*

Among the recently proposed *specifics* for *Diphtheria* we notice, next to lime-water, ranked *lacticacid*, applied in solution by pencil, injection, or atomizer, so as to saturate the false membrane. Dr. Bonaston, in *Union Medicale*, especially commends, on *rational* grounds, the *flowers of sulphur* administered internally, as both curative and prophylactic. Constantin Paul (*Gazette Medicale*) urges *cubebs*, on the authority of M. Trideau, as "precious drug" in this "terrible disease. He employs the extract:

℞. Ext. Cubeb., p. 1 3;

Pulv. Sugar, p. 7;

Pulv. Gum, p. 2.

*M*—Each teaspoonful weighs thirty-eight grains, and to a child four teaspoonfuls may be given daily, dissolved in two or three teaspoonfuls of water. Locally, perchloride of ron.

